# Regorafenib in Glioblastoma Recurrence: How to Deal With MR Imaging Treatments Changes

**DOI:** 10.3389/fradi.2021.790456

**Published:** 2022-02-25

**Authors:** Simona Gaudino, Giammaria Marziali, Carolina Giordano, Riccardo Gigli, Giuseppe Varcasia, Francesca Magnani, Silvia Chiesa, Mario Balducci, Alessandro Maria Costantini, Giuseppe Maria Della Pepa, Alessandro Olivi, Rosellina Russo, Cesare Colosimo

**Affiliations:** ^1^Department of Diagnostic Imaging, Oncological Radiotherapy and Hematology, Institute of Radiology, Fondazione Policlinico Universitario Agostino Gemelli IRCCS, Rome, Italy; ^2^Università Cattolica Sacro Cuore of Rome, Rome, Italy; ^3^Department of Diagnostic Imaging, Oncological Radiotherapy, and Hematology, UOC di Radioterapia Oncologica, Fondazione Policlinico Universitario A. Gemelli IRCCS, Rome, Italy; ^4^Institute of Neurosurgery, Fondazione Policlinico Universitario Agostino Gemelli IRCCS, Catholic University, Rome, Italy

**Keywords:** regorafenib, glioblastoma, MRI, treatment changes, imaging

## Abstract

The treatment of recurrent high-grade gliomas remains a major challenge of daily neuro-oncology practice, and imaging findings of new therapies may be challenging. Regorafenib is a multi-kinase inhibitor that has recently been introduced into clinical practice to treat recurrent glioblastoma, bringing with it a novel panel of MRI imaging findings. On the basis of the few data in the literature and on our personal experience, we have identified the main MRI changes during regorafenib therapy, and then, we defined two different patterns, trying to create a simple summary line of the main changes of pathological tissue during therapy. We named these patterns, respectively, pattern A (less frequent, similar to classical progression disease) and pattern B (more frequent, with decreased diffusivity and decrease contrast-enhancement). We have also reported MR changes concerning signal intensity on T1-weighted and T2-weighted images, SWI, and perfusion imaging, derived from the literature (small series or case reports) and from our clinical experience. The clinical implication of these imaging modifications remains to be defined, taking into account that we are still at the dawn in the evaluation of such imaging modifications.

## Introduction and Background

Glioblastoma (GB), previously called glioblastoma multiforme, is the most common malignant primary brain tumor in adults. Despite multimodality treatment comprising maximal safe resection, radiotherapy, and concomitant and adjuvant chemotherapy, the best median survival is in the range of 14 and 18 months and relapse occurs between 6 and 9 months in over 75% of patients (1). Indeed, conventional therapy has been supported by novel strategies as immunomodulators, immunotherapy, peptide, and mRNA vaccines (2, 3).

From the histological standpoint, GBs are infiltrating glial tumors, displaying abnormal glial cells with variable morphology, high mitotic activity, microvascular proliferation, and necrosis with pseudopalisading patterns. Microvascular proliferation and necrosis are two critical histologic features used for the differentiation between an anaplastic astrocytoma, WHO grade III, and a GB, WHO grade IV. Early clinicopathological studies demonstrated that the degree of microvascular proliferation, as a surrogate of tumor-driven neo-angiogenesis, correlated with survival in patients with high-grade glial tumors (4).

Antiangiogenic approaches have been investigated in both primary and recurrent GB (2, 3, 5, 6) for recurrent GB bevacizumab (BEV) first and, more recently, regorafenib (REG) has been the most studied agents. REG is an oral multi-kinase inhibitor targeting VEGFR-1,−2,−3, tyrosine kinase with Ig and EGF (TIE2), platelet-derived growth factor receptors (PDGFR), Fibroblast growth factor receptors (FGFR), proto-oncogene receptor tyrosine kinase (KIT), Raf-1 Proto-Oncogene, Serine/Threonine Kinase (RAF-1), rearranged during transfection (RET), and BRAF, investigated in the randomized phase II trial Regorafenib in Relapsed Glioblastoma (REGOMA) and approved for the management of recurrent GB by the European Medicines Agency. Since then, the number of patients receiving this agent is increasing, even outside trials, and few single-center experiences and case reports of REG treatment in recurrent GB have been published (7). Moreover, MRI modifications during REG are not yet well codified, and evaluation of GB response could not be straightforward, even for experienced neuroradiologists. Therefore, we provide insights of MRI REG-related changes in recurrent GB.

## Antiangiogenic Therapy, Current Guideline

The optimal treatment of recurrent GB remains controversial, and options include surgery, re-irradiation, and systemic therapy, alone or in combination (1, 8). Surgery for local recurrence is reasonable followed by a second-line treatment. Re-irradiation may be considered in selected cases, and there is no recommended dose or type of radiation used in this setting. Temozolamide (TMZ) is the preferred chemotherapy option if there has been a long interval between the end of adjuvant TMZ and development of recurrent disease, particularly in patient whose tumor is 06-methylguanine-DNA methyltransferase (MGMT)-methylated. Nitrosourea-based treatments, such as carmustina and lomustine, have been widely used in tumor progression and as control in several studies. In 2009, the US Food and Drug Administration approved the use of BEV for the treatment of recurrent GB (8). BEV is a humanized monoclonal antibody directed to the isoform A of the vascular endothelial growth factor (VEGF) (9). Its therapeutic effect is blocking the process of angiogenesis, one of the main features of GB pathogenesis. Most recently, another anti-angiogenic, namely, REG, that has shown efficacy in several cancers (10, 11), as well as preclinical glioma models (12), has been introduced in clinical practice. REG is a multikinase inhibitor that inhibits, among others, the VEGF receptors 1–3 (5). Tyrosine kinases (TKs) are multiple membrane-bound and intracellular kinases that are involved in normal cellular functions. Deregulated action of TKs plays a relevant role in pathologic conditions as cancer. In *in vitro* biochemical or cellular assays, REG or its major human active metabolites has shown efficacy in inhibition of kinases that are very active in angiogenesis (VEGFR1, VEGFR2, VEGFR3, and TIE2), cancer development and growth (KIT, RAF-1, BRAF, and BRAFV600E), and sustaining the tumor microenvironment (PDGFR-alpha, PDGFR-beta, FGFR1, and FGFR2) (13). In *in vivo* models, REG demonstrated anti-angiogenic activity, inhibition of tumor growth, and metastasis (12, 14). In the phase 2 REGOMA trial for first recurrence of a GB, REG increased the median OS from 5.6–7.4 months compared to lomustine (15), with acceptable toxicity and treatment-related adverse events (56 and 40%). The 12-month Overall survival (OS) in the REG group was twice that reported in the lomustine group with a substantial and clinically meaningful reduction in the risk of death.

## The RANO Criteria and the Concept of Pseudo-Response

The response assessment in neuro-oncology (RANO) criteria standardizes the radiologic assessment of treatment response in patients with GB, but they focus primarily on measurements of contrast enhancement (CE), whereas the importance of non-enhancing components of tumors is frequently overlooked (16). Weaknesses in these criteria have emerged with the introduction in the clinical practice of anti-angiogenic drugs. Their main effects of stabilizing the immature and friable vasculature of the tumor and decreasing of rate of microvascular proliferation and the blood-brain barrier permeability translate into a dramatic reduction in the tumor CE as well as reduction of edema on MRI (17, 18). The initial interpretation of tumor response was not confirmed in two different clinical trials (19, 20), and the term pseudoresponse was designated to describe the decrease of the CE on MRI as the effect of the antiangiogenic treatment without a true antitumor effect (21). Regarding BEV, several articles have reported the changes induced by the drug on morphological sequences, as well as on diffusion-weighted imaging (DWI) and perfusion-weighted imaging (PWI), also assessing the association between MRI pattern and patient survival. Less well-known is the effect of REG on morphological and non-morphological MRI sequences. Knowing the different ways of action of the two drugs, it is inductive to hypnotize that drug-induced MRI changes may be similar but not the same.

## MRI Changes in Regorafenib Treatment

We propose two patterns of MRI changes in GB recurrence under BEV treatment (patterns A and B), comparing the MRI immediately prior to REG onset and the next MRI.

To summarize these pattern, we relied on the literature review (reported below in the text), and on our personal experience based on 28 patients with recurrent GB. They were all isocitrate dehydrogenase (IDH) wild type, and 11/28 methylation of MGMT promoter was present. All patients received postoperative RT in combination with TMZ, according to the STUPP regimen, and received REG as a last line treatment. MRI exams had been acquired almost exclusively on 1.5T scanners, with highly variable protocols (having been acquired in different canters), and were evaluated by neuroradiologists with experience in neuro-oncology.

To sum up the two patterns, we assessed morphological sequences as T2, FLAIR, T1, and T1 after gadolinium, as well as DWI and apparent diffusion coefficient (ADC) map, susceptibility-weighted imaging (SWI), and PWI, the latter nowadays are part of many clinical standard glioma protocols.

Pattern A is similar to the classic progression disease model, reported by increasing CE and increasing T2/FLAIR signal abnormality.

Pattern B holds MRI changes frequently reported as T2-dominant growth, characterized by decreasing CE and increasing (relative or absolute) T2/FLAIR hyperintensity. Pattern B also includes some MRI findings reported by case reports that, based on our experience, are more associated with this pattern (23, 24).

### Pattern A

**Figure 1:**
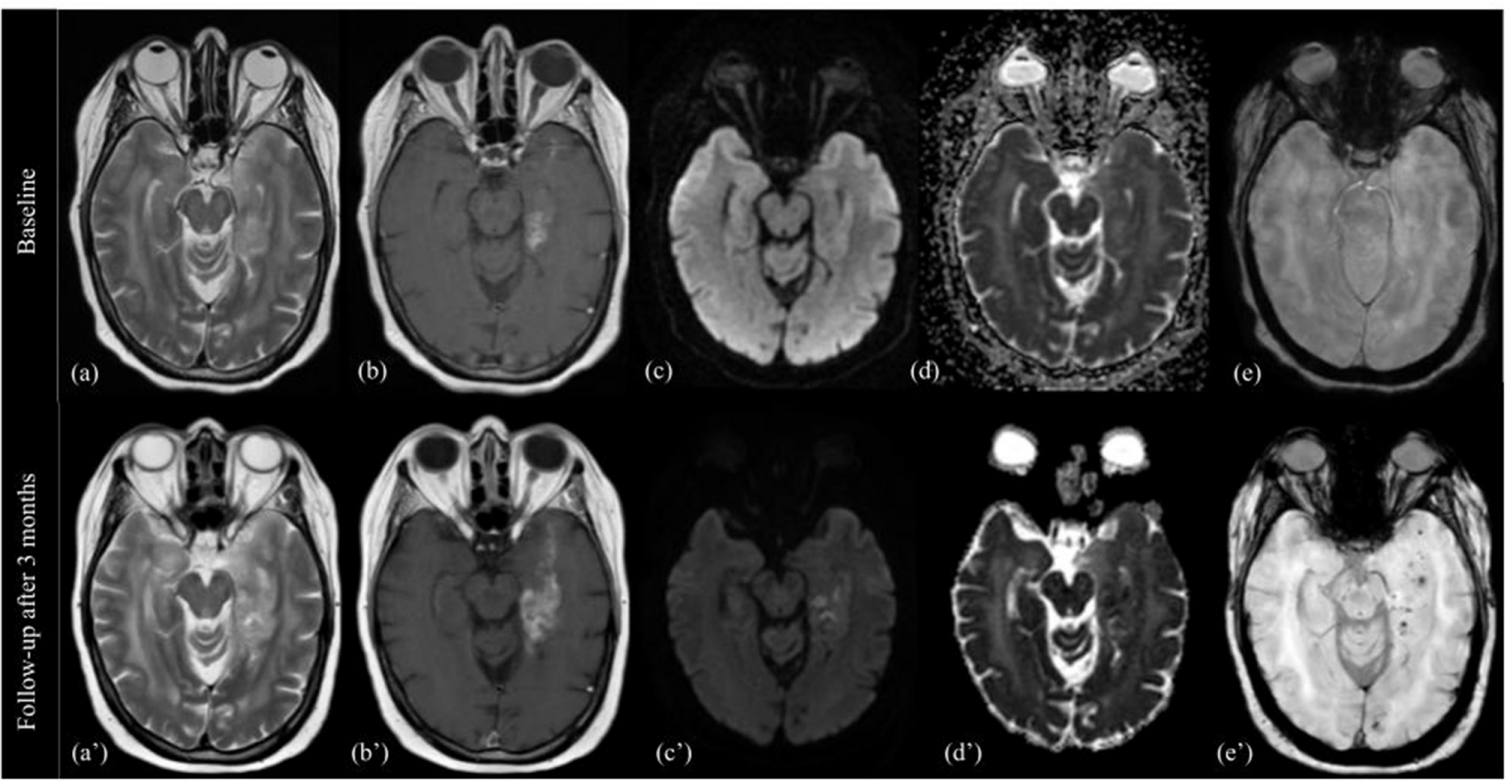
Pattern A. MRI changes in recurrent high grade glioma under regorafenib, pattern A. MRI scans performed at baseline (top image) and 3 months after first administration of REG therapy (bottom image). T2-weighted images **(a,a')**, T1-weighted images with gadolinium **(b,b')**, DWI images **(c,c')**, apparent diffusion coefficient **(d,d')**, and SWI images **(e,e')** are shown from left to right. The 3-month follow-up showed increase in size, more than 25%, of enhancing tumor in the left temporo-mesial area, and progressive extension into the ipsilateral parahippocampal gyrus. T2 signal intensity was similar to the previous MR exam, but contained focal hyperintensities on DWI (corresponding to low ADC values), and multiple black dots on SWI.

T2/FLAIR: there are no evident modifications of the signal intensity in the solid components of the tumor, the T2 signal intensity, already heterogeneous, can increase focally or diffusely, mixed with a component of mild hypointensity, and already evident in previous MRI (Figure 1).

DWI/ADC: stable or new sporadic hyperintensity dots.

T1: usually iso-hypointense, without evidence of hyperintensities in the tumor area.

SWI: black dots within the solid component.

T1 after contrast: Pattern of CE similar to previous MRI exam, usually increase in size/extension.

PWI: high rCBV, somewhat stable or increase compared to previous MRI.

Edema: increase.

### Pattern B

**Figure 2:**
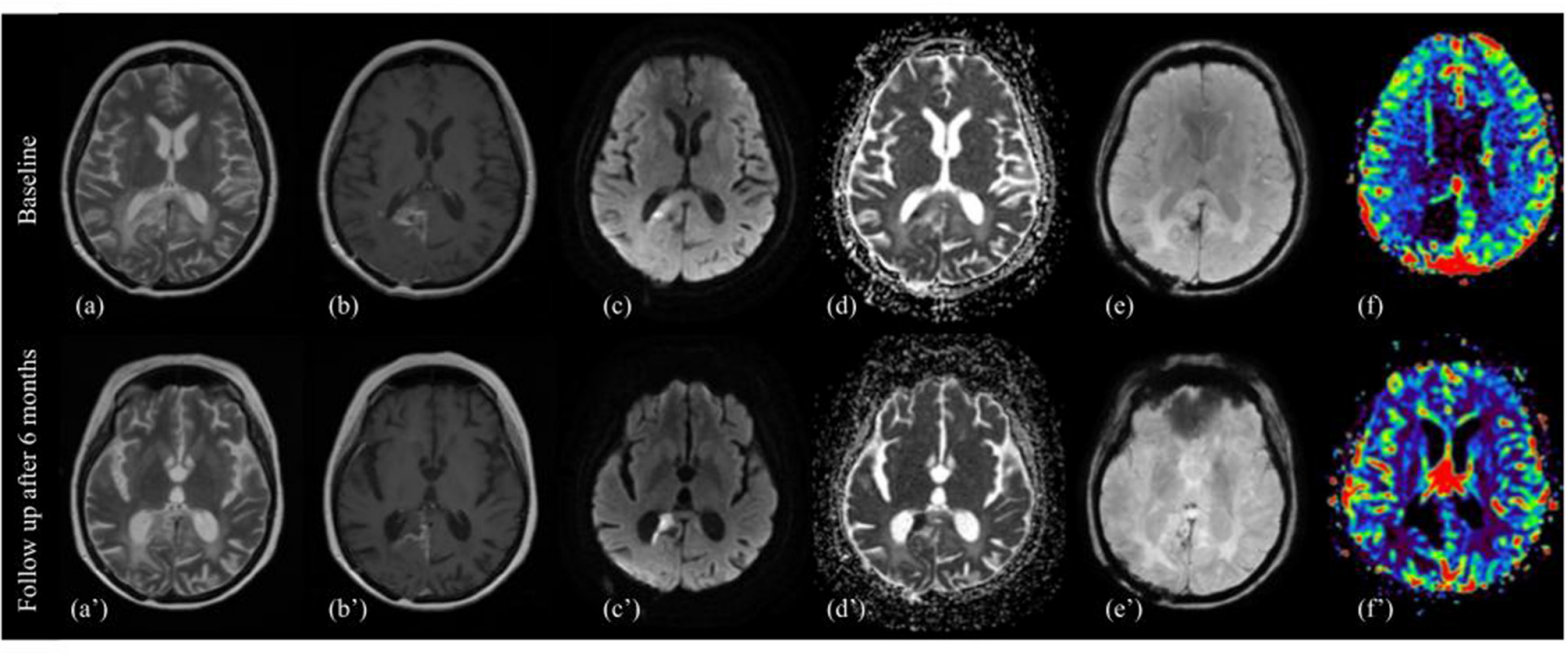
Pattern B. MRI changes in recurrent high grade glioma under regorafenib, pattern B. MRI scans performed at baseline (top image) and after six months (bottom image) from the first administration of REG therapy. T2w images **(a,a')**, contrast enhanced T1w images **(b,b')**, diffusion-weighted b1000 **(c,c')**, apparent diffusion coefficients (ADC) map **(d,d')**, SWI images **(e,e')**, and color-coded perfusion map **(f,f')** are shown from left to right. On 6-month follow-up MRI, the previously enhanced tumor component showed a dramatically absence of CE, diffusion restriction on DWI and ADC map, and decrease signal on corresponding T2 image, surrounding by a thin hypointense rim on SWI, and peripheral contrast enhancement on T1-wi after gadolinium. There was also a decrease of peritumoral edema.

T2/FLAIR: increase in the T2 component, relative to the reduction of the enhancing component, and often absolute for a significant increase of T2 abnormality compared to pre-Reg MRI. However, we noticed that, under a careful evaluation of the T2-w images, tumor components that decrease contrast enhancement also decrease T2 signal intensity, with better differentiation from the hyperintense perifocal edema (Figure 2).

DWI/ADC: marked hyperintensity on DWI (with equally marked hypointensity on ADC map) of tumor components showing decreased signal intensity on T2.

T1: no visually noticeable changes.

SWI: hypointense rim surrounding the hyperintense tissue on DWI, smooth, and both complete and incomplete, often corresponding to marginal enhancement on T1 after gadolinium.

T1 after contrast: overall decrease of contrast enhancement of the target lesions, with marginal or dot-like enhancing component, residual or of new-onset.

PWI: reduced relative cerebral blood volume (rCBV) in the DWI hyperintensity component. Outside the DWI hyperintensity usually decrease or just mild increase of rCBV. There are, however, reported cases of increased rCBV (22, 23).

An overall decrease of the peritumoral edema, without an increase of steroid dosage, was observed in some patients.

## Discussion

The treatment of recurrent gliomas remains a major challenge of daily neuro-oncology practice, and imaging findings of new therapies may be challenging. Novel therapies have led to the occurrence of interesting but sometimes confusing post-treatment imaging appearances, as happened with BEV treatment and the coining of the term pseudoresponse. At first, MRI changes during BEV had puzzled neuroradiologists, as they presented differently from those observed during other treatments in several MRI sequences. The first step was to catalog the new MRI changes and then attempt to describe radiographic patterns of MRI changes and to correlate with outcome. Previous studies have shown that most significant modifications during BEV concerned proton diffusivity, CE, and T1 signal intensity evaluated with qualitative or quantitative methods. Therefore, specific MRI patterns including ADC hypointensity (in terms of visual signal intensity and ADC histograms), presence of T1 hyperintensity, and changes in T1 enhancing volume were proposed as radiographic prognostic models (25).

**Figure 3:**
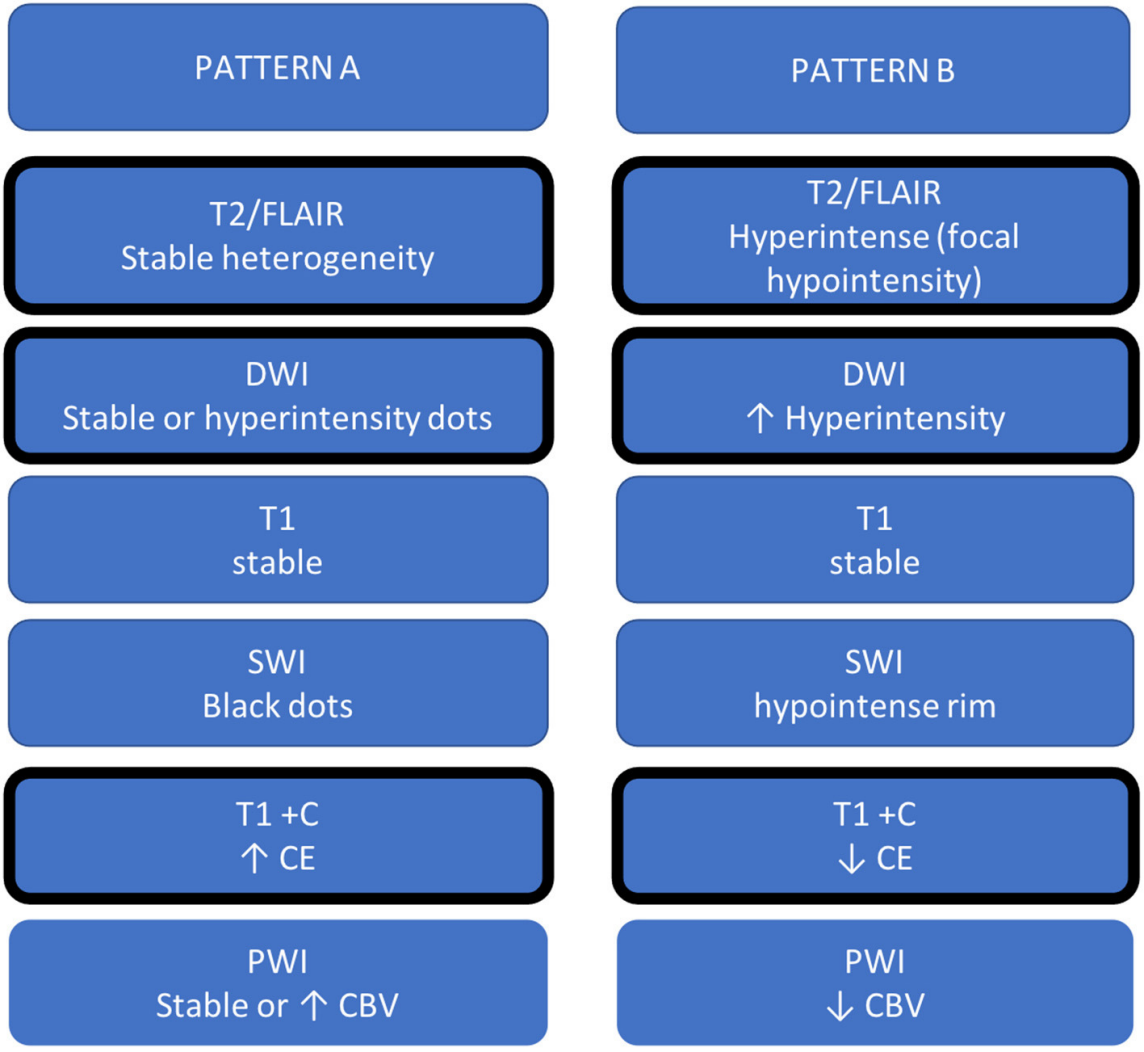
Summary table of MRI changes during REG. The patterns defined as A and B are divided into two columns, and the MRI sign changes most frequently reported in the literature have been graphically identified with a thicker cell margin.

Recently, few studies reported MRI changes during REG therapy, somewhat similar to what was observed for BEV, and a “T2-dominant growth pattern,” a term coined for BEV, was also associated with REG (26). However, REG studies were based on a small cohort and a few MRI sequences. From the literature review (including case reports) and based on our clinical experience, we reported two MRI progression patterns, containing more sequences as DWI, SWI, and PWI, and named patterns A and B. Figure 3 summarizes A and B patterns.

**Pattern A** is similar to classic progression disease.

There is a trend toward an increase in **T2/FLAIR** signal intensity, presumed to represent edema and tumor infiltration growth (27).

**DWI** changes appear less pronounced than in pattern B, with new or increased small areas of hyperintensity, not always corresponding to less diffusivity on ADC maps. As previously mentioned, focal restricted diffusion may correlate with increased tumor cellularity, as well as with ischemia, or other treatment changes (cell death, necrosis, and hemorrhage), which affect the Brownian movement of water (28).

On **SWI**, there are an increasing number of the so-called intratumoral black dots, intratumoral susceptibility signals, or SWI-positive tumor pixels (29), which have been proposed as an early biomarker of tumor progression for GB treated with postoperative chemo-radiotherapy (TMZ) (30).

On **T1-w after gadolinium administration**, CE areas usually increase in size/extension. It has been well established that CE most often corresponds to the highest density of tumor tissue and the most aggressive histological features in gliomas (31).

**Perfusion MRI** can be used to image neovascularization, as a hallmark of tumor progression. The abnormal vascular proliferation increases the amount of blood per brain tissue volume unit, and, consequently, relative CBV was significantly higher in patients with recurrent GB (32, 33).

**Pattern B** includes most features of the “T2-dominant growth pattern.” In particular, the main finding in pattern B was the marked hyperintensity of signal on DWI, corresponding to low signal intensity on T2-wi, and marked decrease of CE. This pattern was similar to what was described for BEV treatment and referred to as “stroke-like” DWI restriction. However, although DWI hyperintensity in BEV was (pathologically) reported as coagulative necrosis surrounded by the viable hypercellular tumor (34), a precise histological correlation and interpretation of modification under REG is still uncertain. Mansour et al. (23) hypothesized that the diffusion restriction could be explained by constant hemorrhagic diapedesis. In addition, the reduction of CE has already been associated with antiangiogenic agents (35).

Regarding the hypointense rim on SWI, it was thinner and less marked, as reported by Mansour et al. on the first MRI after REG, more similar to what was demonstrated in the subsequent follow-up MRI (23). The black rim on SWI has been described in many brain diseases, as in cerebral abscesses, GB, and progressive multifocal leukoencephalopathy, respectively, representing granulation tissue, blood product, or neuroinflammatory process (36, 37). We do not currently know the significance of this hypointensity, but we may postulate that it may be due to one or a combination of the mentioned above causes, as well as to free radicals or other causes of susceptibility. In pattern B, predominantly observed PWI modification was hypoperfusion. As reported for BEV, decreased perfusion was probably due to the antiangiogenetic action that prevents the development of a rich vascular system with regression of hypervascularization in the tumor (38).

Although recent studies indicate poor performance of regorafenib in recurrent high-grade glioma (39), it may be potential for clinical utility to understand whether the two MRI patterns may correlate with different survival. Taking into account the current relative lack of REG cases reported in the literature, it will probably take several studies to identify and categorize radiographic patterns to respond to REG, as happened with BEV (40). Certainly, more extensive researches on a larger patient cohort are necessary, as well as the combination of a large number of MRI data coming from morphological and non-morphological sequences (DWI, PWI, and MRS) (41), also considering the opportunity of processing multi-parametric data through deep learning to arrive at more definite conclusions (42).

## Conclusion

MRI assessment in recurrent GB treated with REG remains a challenge, and radiologists have to be aware of these new post treatment imaging. A more extensive and rigorous collection and assessment of multiparametric MR data and identification of MRI patterns correlated with clinical outcome are needed to overcome this challenge.

## Author Contributions

SG gives substantial contributions to the conception and design of the work, analysis and interpretation of data for the work. GM, CG, RG, GV, FM, SC, MB, AC, RR, and CC give substantial contributions to the acquisition, analysis and interpretation of data for the work. All authors contributed to the article and approved the submitted version.

## Conflict of Interest

The authors declare that the research was conducted in the absence of any commercial or financial relationships that could be construed as a potential conflict of interest.

## Publisher's Note

All claims expressed in this article are solely those of the authors and do not necessarily represent those of their affiliated organizations, or those of the publisher, the editors and the reviewers. Any product that may be evaluated in this article, or claim that may be made by its manufacturer, is not guaranteed or endorsed by the publisher.

## References

[1] Stupp R Mason WP van den Bent MJ Weller M Fisher B Taphoorn MJ. Radiotherapy plus concomitant and adjuvant temozolomide for glioblastoma. N Engl J Med. (2005) 352:987–96. 10.1056/NEJMoa043330 15758009

[2] Clavreul A Pourbaghi-Masouleh M Roger E Menei P. Nanocarriers and nonviral methods for delivering antiangiogenic factors for glioblastoma therapy: the story so far. Int. J. Nanomed. (2019) 14:2497–2513. 10.2147/IJN.S194858 31040671 PMC6461002

[3] Gerstner ER Batchelor TT. Antiangiogenic therapy for glioblastoma. Cancer J. (2012) 18:45–50. 10.1097/PPO.0b013e3182431c6f 22290257 PMC3269655

[4] Folkerth RD. Descriptive analysis and quantification of angiogenesis in human brain tumors. J Neurooncol. (2000) 50:165–72. 10.1023/a:1006499824379 11245275

[5] Wilhelm Scott M Dumas J Adnane L Lynch M Carter CA Schütz G. Regorafenib (BAY 73-4506): a new oral multikinase inhibitor of angiogenic, stromal and oncogenic receptor tyrosine kinases with potent preclinical antitumor activity. Int J Cancer. (2011) 129:245–55. 10.1002/ijc.25864 21170960

[6] Anthony C Mladkova-Suchy N Adamson D.C. The evolving role of antiangiogenic therapies in glioblastoma multiforme: current clinical significance and future potential. Expert Opin. Investig. Drugs. (2019) 28:787–97. 10.1080/13543784.2019.1650019 31356114

[7] Glas M Kebir S. Regorafenib in glioblastoma recurrence: how to deal with conflicting ‘real-life’ experiences? Ther Adv Med Oncol. (2019) 11:1758835919887667. 10.1177/1758835919887667 31897090 PMC6918487

[8] Cohen MH Shen YL Keegan P Pazdur R. FDA drug approval summary: bevacizumab (Avastin) as treatment of recurrent glioblastoma multiforme. Oncologist. (2009) 14:1131–8. 10.1634/theoncologist.2009-0121 19897538

[9] Gerstner ER Duda DG Di Tomaso E Ryg PA Loeffler JS Sorensen AG. VEGF inhibitors in the treatment of cerebral edema in patients with brain cancer. Nat Rev Clin Oncol. (2009) 6:229–36. 10.1038/nrclinonc.2009.14 19333229 PMC4793889

[10] Grothey A Van Cutsem E Sobrero A Siena S Falcone A Ychou M. Regorafenib monotherapy for previously treated metastatic colorectal cancer (CORRECT): an international, multicentre, randomised, placebo-controlled, phase 3 trial. Lancet. (2013) 381:303–12. 10.1016/S0140-6736(12)61900-X 23177514

[11] Bruix J Qin S Merle P Granito A Huang YH Bodoky G. Regorafenib for patients with hepatocellular carcinoma who progressed on sorafenib treatment (RESORCE): a randomised, double-blind, placebo-controlled, phase 3 trial. Lancet. (2017) 389:56–66. 10.1016/S0140-6736(16)32453-9 27932229

[12] Hamed HA Tavallai S Grant S Poklepovic A Dent P. Sorafenib/regorafenib and lapatinib interact to kill CNS tumor cells. J. Cell Physiol. (2015) 230:131–9. 10.1002/jcp.24689 24911215 PMC4182138

[13] Goel G. Evolution of Regorafenib from bench to bedside in colorectal cancer: Is it an attractive option or merely a “me too” drug? Cancer Manag Res. (2018) 10:425–37. 10.2147/CMAR.S88825 29563833 PMC5844550

[14] Fondevila F Méndez-Blanco C Fernández-Palanca P González-Gallego J Mauriz JL. Anti-tumoral activity of single and combined regorafenib treatments in preclinical models of liver and gastrointestinal cancers. Exp Mol Med. (2019) 51:1–15. 10.1038/s12276-019-0308-1 31551425 PMC6802659

[15] Lombardi G De Salvo GL Brandes AA Eoli M Rudà R Faedi M. Regorafenib compared with lomustine in patients with relapsed glioblastoma (REGOMA): a multicentre, open-label, randomised, controlled, phase 2 trial. Lancet Oncol. (2019) 20:110–9. 10.1016/S1470-2045(18)30675-2 30522967

[16] Eisele SC Wen PY Lee EQ. Assessment of brain tumor response: RANO and its offspring. Curr Treat Options Oncol. (2016) 17:35. 10.1007/s11864-016-0413-5 27262709

[17] Hasselbalch B Lassen U Hansen S Holmberg M Sørensen M Kosteljanetz M. Cetuximab, bevacizumab, and irinotecan for patients with primary glioblastoma and progression after radiation therapy and temozolomide: a phase II trial. Neuro Oncol. (2010) 12:508–16. 10.1093/neuonc/nop063 20406901 PMC2940618

[18] Vredenburgh JJ Desjardins A Herndon JE Marcello J Reardon DA Quinn JA. Bevacizumab plus irinotecan in recurrent glioblastoma multiforme. J Clin Oncol. (2007) 25:4722–9. 10.1200/JCO.2007.12.2440 17947719

[19] Gilbert MR Dignam JJ Armstrong TS Wefel JS Blumenthal DT Vogelbaum MA. A randomized trial of bevacizumab for newly diagnosed glioblastoma. N Engl J Med. (2014) 370:699–708. 10.1056/NEJMoa1308573 24552317 PMC4201043

[20] Chinot OL Wick W Mason W Henriksson R Saran F Nishikawa R. Bevacizumab plus radiotherapy-temozolomide for newly diagnosed glioblastoma. N Engl J Med. (2014) 370:709–22. 10.1056/NEJMoa1308345 24552318

[21] Brandsma D van den Bent MJ. Pseudoprogression and pseudoresponse in the treatment of gliomas. Curr Opin Neurol. (2009) 22:633–8. 10.1097/WCO.0b013e328332363e 19770760

[22] Zeiner P.S Kinzig M Div'e I Maurer G.D Filipski K Harter PN. Regorafenib CSF penetration, efficacy, and MRI patterns in recurrent malignant Glioma patients. J Clinical Med. (2019) 8:2031. 10.3390/jcm8122031 31766326 PMC6947028

[23] Mansour M. Modification of MRI pattern of high-grade glioma pseudoprogression in regorafenib therapy. J Med Imaging Radiat Oncol. (2021). 10.1111/1754-9485.13267. [Epub ahead of print]. 34169667

[24] Detti B Scoccianti S Lucidi S Maragna V Teriaca MA Ganovelli M. Regorafenib in glioblastoma recurrence: A case report. Cancer Treat Res Commun. (2021) 26:100263. 10.1016/j.ctarc.2020.100263 33338858

[25] Cachia D Nabil AE Kamiya-Matsuoka C Masumeh H Alfaro-Munoz KD Mandel J. Radiographic patterns of progression with associated outcomes after bevacizumab therapy in glioblastoma patients. J Neurooncol. (2017) 135:75–81. 10.1007/s11060-017-2550-5 28702781

[26] Gatto L Franceschi E Tosoni A Di Nunno V Maggio I Tonon C. Distinct MRI pattern of “pseudoresponse” in recurrent glioblastoma multiforme treated with regorafenib: case report and literature review. Clin Case Rep. (2021) 9:e04604. 10.1002/ccr3.4604 34457284 PMC8380081

[27] Watanabe M Tanaka R Takeda N. Magnetic resonance imaging and histopathology of cerebral gliomas. Neuroradiology. (1992) 34:463–9. 10.1007/BF00598951 1436452

[28] Le Fèvre C Constans JM Chambrelant I Antoni D Bund C Leroy-Freschini B. Pseudoprogression versus true progression in glioblastoma patients: a multiapproach literature review. Part 2-Radiological features and metric markers. Crit Rev Oncol Hematol. (2021) 159:103230. 10.1016/j.critrevonc.2021.103230 33515701

[29] Hsu CC Watkins TW Kwan GN Haacke EM. Susceptibility-weighted imaging of glioma: update on current imaging status and future directions. J Neuroimaging. (2016) 26:383–90. 10.1111/jon.12360 27227542

[30] van Leyen K Roelcke U Gruber P Remonda L Berberat J. Susceptibility and tumor size changes during the time course of standard treatment in recurrent glioblastoma. Neuroimaging. (2019). 29:645–9. 10.1111/jon.12631 31112344

[31] Ellingson BM Wen PY Cloughes TF. Evidence and context of use for contrast enhancement as a surrogate of disease burden and treatment response in malignant glioma. Neuro Oncol. (2018) 20:457–71. 10.1093/neuonc/nox193 29040703 PMC5909663

[32] Barajas RF Jr Chang JS Segal MR Parsa AT McDermott MW Berger MS. Differentiation of recurrent glioblastoma multiforme from radiation necrosis after external beam radiation therapy with dynamic susceptibility-weighted contrast-enhanced perfusion MR imaging. Radiology. (2009) 253:486–96. 10.1148/radiol.2532090007 19789240 PMC2770116

[33] van Dijken BRJ BSc van Laar PJ Smits M Dankbaar JW Enting RH. Perfusion MRI in treatment evaluation of glioblastomas: clinical relevance of current and future techniques. J Magn Reson Imaging. (2019) 49:11–22. 10.1002/jmri.26306 30561164 PMC6590309

[34] Nguyen HS Milbach N Hurrell SL Cochran E Connelly J Bovi JA. Progressing bevacizumab-induced diffusion restriction is associated with coagulative necrosis surrounded by viable tumor and decreased overall survival in patients with recurrent glioblastoma. AJNR Am J Neuroradiol. (2016) 37:2201–8. 10.3174/ajnr.A4898 27492073 PMC5161572

[35] Huang RY Neagu MR Reardon DA Wen PY. Pitfalls in the neuroimaging of glioblastoma in the era of antiangiogenic and immuno/targeted therapy-detecting illusive disease, defining response. Front Neurol. (2015) 6:33. 10.3389/fneur.2015.00033 25755649 PMC4337341

[36] Lai PH Chung HW Chang HC Fu JH Wang PC Hsu SH. Susceptibility-weighted imaging provides complementary value to diffusion-weighted imaging in the differentiation between pyogenic brain abscesses, necrotic glioblastomas, and necrotic metastatic brain tumors. Eur J Radiol. (2019) 117:56–61. 10.1016/j.ejrad.2019.05.021 31307653

[37] Thurnher MM Boban J Rieger A Gelpi E. Susceptibility-Weighted MR Imaging hypointense rim in progressive multifocal leukoencephalopathy: the end point of neuroinflammation and a potential outcome predictor. AJNR Am J Neuroradiol. (2019) 40:994–1000. 10.3174/ajnr.A6072 31122919 PMC7028608

[38] Pope WB. Predictive imaging marker of bevacizumab efficacy: perfusion MRI. Neuro Oncol. (2015) 17:1046–7. 10.1093/neuonc/nov067 25910842 PMC4490878

[39] Kebir S Rauschenbach L Radbruch A Lazaridis L Schmidt T Stoppek A-K. Regorafenib in patients with recurrent high-grade astrocytoma. J Cancer Res Clin Oncol. (2019) 145:1037–42. 10.1007/s00432-019-02868-5 30820715 PMC11810342

[40] Nowosielski M Ellingson BM Chinot OL Garcia J Revil C Radbruch A. Radiologic progression of glioblastoma under therapy-an exploratory analysis of AVAglio. Neuro Oncol. (2018) 20:557–66. 10.1093/neuonc/nox162 29016943 PMC5909665

[41] Li C Gan Y Chen H Chen Y Deng Y Zhan W. Advanced multimodal imaging in differentiating glioma recurrence from post-radiotherapy changes. Int Rev Neurobiol. (2020) 151:281–97. 10.1016/bs.irn.2020.03.009 32448612

[42] Lee J Wang N Turk S Mohammed S Lobo R Kim J. Discriminating pseudoprogression and true progression in diffuse infiltrating glioma using multi-parametric MRI data through deep learning. Arvind Rao Sci Rep. (2020) 10:20331. 10.1038/s41598-020-77389-0 33230285 PMC7683728

